# Moderator role of Type D personality traits between depressive symptoms and job satisfaction among teachers

**DOI:** 10.3389/fpubh.2024.1402422

**Published:** 2024-05-03

**Authors:** Ayşegül Yetkin Tekin, Hekim Karadağ

**Affiliations:** ^1^Education Faculty, Psychological Counseling and Guidance Department, Adıyaman University, Adıyaman, Türkiye; ^2^Psychology Department, Van Education and Research Hospital, Van, Türkiye

**Keywords:** depressive symptoms, job satisfaction, negative affect, social inhibition, teachers, Type D personality

## Abstract

**Background:**

Type D personality is characterized by negative affect (NA) and social suppression (SI). It has been indicated Type D personality is associated with depression, anxiety, and burnout. Depressive complaints and social inhibition negatively affect job satisfaction. The aim of this study is to investigate the moderating role of Type D personality structure between the severity of depressive complaints and job satisfaction in teachers.

**Methods:**

939 teachers, who constitute the sample of the study, completed the sociodemographic form, Type D personality scale (DS-14), Beck Depression Inventory (BDI) and Minnesota Satisfaction Scale Short Form with an online survey.

**Results:**

While a negative relationship was found between teachers’ NA scores and their intrinsic and extrinsic job satisfaction (*r* = −0.28 and *r* = −0.19, respectively), a negative relationship was detected between SI scores and intrinsic and extrinsic job satisfaction (*r* = −0.22 and *r* = −0.21, respectively). NA and SI had partial moderating roles in the relationship between BDI score and intrinsic job satisfaction. SI played a partial moderating role in the relationship between BDI and extrinsic job satisfaction.

**Conclusion:**

It can be said Type D personality traits has a moderating role between the severity of teachers’ depressive complaints and job satisfaction.

## Introduction

Type D personality describes the personality characteristics of individuals prone to psychological stress and has two substructures: negative affect and social inhibition (1). While negative affect is characterized by the predominance and longer duration of unwanted negative emotions, social inhibition represents the reduction of social interaction and suppression of the expression of emotions (2). It has been reported that individuals with Type D personality may have a more depressed and dysphoric affect and have anxiety about rejection and disapproval from others (3, 4). Some studies have shown that individuals with Type D personality may be more prone to both stress-related physical problems such as cardiovascular diseases and psychological problems such as depression, burnout, and anxiety disorders (5–10).

Job satisfaction is defined as the individual’s positive feelings about his or her profession or job, as well as his or her evaluation of how the profession or job meets the individual’s needs, desires, and expectations (11). Job satisfaction can be affected by individual and job-related variables. Many studies have shown that job satisfaction can be affected by factors related to the job performed. For example, it is known that working hours, income, supervision, career goals, and relationships in the work environment have an impact on job satisfaction (12–15).

On the other hand, job satisfaction may also be affected by individual characteristics. For example, it has been reported that personality structure or characteristics may affect job satisfaction (16, 17). Personality-specific traits such as extraversion, introversion, and neuroticism have an impact on an individual’s job satisfaction (18, 19). The hypothesis that the type D personality structure may negatively affect job satisfaction due to its main components (NA and SI) has been investigated in some employee groups other than teachers. It has been found that healthcare professionals with a Type D personality have lower job satisfaction than those without a Type D personality, and Type D personality may negatively affect the job satisfaction of nurses (20, 21).

One of the important factors affecting job satisfaction is the psychological problems of the individual. Many studies have shown that psychological factors such as depression, high-stress levels, anxiety disorders, burnout, and sleep problems can negatively affect an individual’s job satisfaction (22–26). Depression is one of the most common mental problems in the world and one that causes the most disability (27). Depression, which is characterized by symptoms such as anhedonia, loss of interest, depressed mood, sleep-appetite problems, loss of energy, and beliefs of worthlessness or low self-esteem, suicidal ideations, has been reported as a factor that negatively affects job satisfaction in many professional groups (28–30). Research in different occupational groups has shown that core symptoms of depression, such as anhedonia, loss of interest, and depressed mood, can reduce job satisfaction (31). It is also known that depressive symptoms can negatively affect job satisfaction in teachers, who are one of the professional groups that work in direct interaction with people (32, 33).

Based on all the information highlighted above, it is clear that there is a relationship between depressive complaints and job satisfaction. On the other hand, it can be said that type D personality, which is characterized by negative affect and social suppression, increases the tendency to some psychological problems such as depressive symptoms, burnout, and anxiety. It can be said that the type D personality structure has components that can affect both the emergence of depressive symptoms and job satisfaction and thus may play a role in the relationship between depressive symptoms and job satisfaction. To our knowledge, there is no research examining the moderating role of Type D personality structure between depressive symptom severity and job satisfaction. The main hypothesis of this study is that the Type D personality structure may have a regulatory function between depressive complaints and job satisfaction due to its defining characteristics (NA and SI). The aim of this study is to investigate the moderating role of Type D personality structure between the severity of depressive symptoms and job satisfaction in teachers.

## Materials and methods

### Participants

The present study had a cross-sectional and descriptive nature. The sample of the research consisted of 939 teachers working in 73 public schools in Van Province and voluntarily filling out the online survey sent with the permission of the Directorate of National Education. 63 of the participants (6.7%) were preschool teachers, 544 were primary school teachers (57.9%), and 332 were high school teachers (35.4%). 761 of the teachers (81%) were working in schools in the city and district centers, and 178 (19%) were working in village schools. The inclusion criteria for the study were being an active teacher, not having a serious psychiatric disease (such as schizophrenia, bipolar disorder, or substance use disorder), and being a volunteer. All stages of the study were approved by the Van Education and Research Hospital Ethics Committee (2022/07–02), and permission was obtained from the National Education Directorates for the study (Permission Number: 49492119). It ensured that there was a research process in accordance with the Declaration of Helsinki and research in humans. The ethics committee legally monitored the online interview technique, data collection, and confidentiality of participants’ data. The consent of the participants was obtained in the online survey application. The first screen of the online survey explained the purpose of the study and asked whether the participant would like to participate in the study voluntarily. The participants who wanted to participate in the research voluntarily could access the next forms only after pressing the approval button.

### Material

#### Sociodemographic form

This form includes participants’ characteristics such as age, gender, marital status, income level, working years, and weekly working hours.

#### Type D personality scale (DS-14)

It is a Likert-type self-report scale consisting of 14 items developed by Denollet (2). The scale consists of two subscales: negative affect and social inhibition. Each item is scored between 0 and 4. While the scores that can be calculated from the subscales (NA and SI) vary between 0 and 28 points, the total score of the scale varies between 0 and 56. Increasing scores from the subscales means that Type D personality traits also increase. Turkish validity and reliability study was conducted by Alçelik et al. (34). Internal consistency coefficients for the Turkish form of the scale are 0.82 for NA and 0.81 for SI.

#### Minnesota satisfaction scale short form (MSS-SF)

It is a Likert-type self-report scale consisting of 20 items developed by Weiss et al. (35). The scale consists of two subscales: intrinsic satisfaction and extrinsic satisfaction. Intrinsic satisfaction consists of factors related to satisfaction with the internal nature of the job, such as success, recognition or acceptance, job responsibility, and change of duties due to promotion. Extrinsic satisfaction consists of elements related to the business environment, such as business dynamics, control style, relations with the manager, work and subordinates, working conditions, and wages. Each item varies between 1 and 5 points. The scale has 12 items that evaluate intrinsic satisfaction and 8 items that evaluate extrinsic satisfaction. As the scores from the subscales increase, job satisfaction also increases. Turkish validity and reliability study was conducted by Baycan (36). In the Turkish validity and reliability study of the scale, the Cronbach’s alpha value was found to be 0.77.

### Statistical analysis

SPSS 25.0 package program and Jamovi 2.4.14 statistical program were used for statistical analysis of the data. Sociodemographic characteristics and the scale scores of the participants were calculated as mean, standard deviation, number and percentage. Normality distribution of continuous variables was examined with the Kolmogorov Smirnov test. Pearson’s correlation test was used to evaluate the relationship between BDI, MSS-SF, and DS-14 scales scores. The role of NA and SI between BDI, and IS and ES was investigated by moderator analysis. Harman’s single-factor test was used to evaluate common method variance bias, and all self-report scale scores used in the study were subjected to non-rotation factor analysis (When the number of factors is limited to 1, the total variance explained is 32.70%, and if the rate was lower than 50%, it was interpreted that there was no common method variance error in the study). Bonferroni’s correction was used in multiple comparisons in order to minimize the risk of type I error. In all analyses, the significance level was accepted as *p* < 0.05.

## Results

The mean age of the teachers was 39.8 ± 6.8 years (Range of age = 26–58 years). 55.6% (*n* = 522) of the teachers were male and 44.4% (*n* = 417) were female. The sociodemographic characteristics and scale scores of the participants were shown in Table 1.

**Table 1:** Characteristics of the teachers.

Variable		Mean ± SD	*N* (%)	Range
Age		39.8 ± 6.8		26–58
Gender	Female		417 (44.4)	
	Male		522 (55.6)	
Marital status	Married		823 (87.6)	
	Single		94 (10)	
	Widow		22 (2.3)	
Income level	Low		83 (8.8)	
	Medium		783 (83.4)	
	High		73 (7.8)	
Job duration (year)		11.3 ± 5.9		2–30
Weekly working time (hour)		19.5 ± 4.7		12–32
BDI score		24.7 ± 13		0–56
Type-D personality	Negative affectivity	10 ± 6.8		0–28
	Social inhibition	12.1 ± 7.1		0–28
Job satisfaction	Internal satisfaction	30.4 ± 11.6		12–60
	External satisfaction	22.6 ± 9.6		8–41

**Figure 1:**
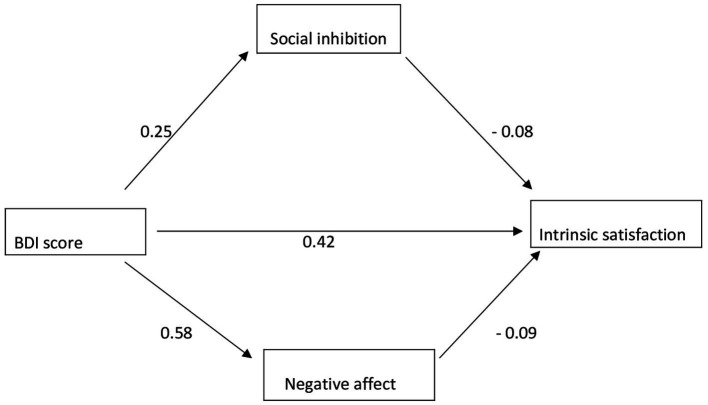
Moderator variable analysis for intrinsic satisfaction.

According to the moderation relationship carried out for the intrinsic satisfaction sub-dimension of job satisfaction, the indirect effect is negative affect (*β* =0.049, SE = 0.018, *p* < 0.001) and social inhibition (*β* =0.021, SE = 0.007, *p* < 0.001). And total effect (*β* =0.421, SE = 0.026, *p* < 0.001) results are significant. Although the direct effect result was significant (*β* =0.350, SE = 0.032, *p* < 0.001), the effect level decreased when compared to the total effect when the moderator variable was included in the model. This result shows that negative affect and social inhibition have a partial moderating role in the relationship between depression and intrinsic job satisfaction. The results are in Figure 1 and Table 2.

**Table 2:** Moderator variable analysis results for intrinsic satisfaction.

Effect	Estimate	SE	*β*	*z*	*p*
BDI ⇒ NA ⇒ IS	−0.0442	0.01870	−0.0498	−2.37	0.018
BDI ⇒ SI ⇒ IS	−0.0189	0.00731	−0.0213	−2.59	0.010
BDI ⇒ NA	0.3006	0.01392	0.5760	21.59	< 0.001
NA ⇒ IS	−0.1472	0.06184	−0.0864	−2.38	0.017
BD ⇒ SI	0.1386	0.01725	0.2537	8.04	< 0.001
SI ⇒ IS	−0.1366	0.04992	−0.0840	−2.74	0.006
BDI ⇒ IS	−0.3115	0.03222	−0.3503	−9.67	< 0.001
BDI ⇒ IS	−0.3747	0.02633	−0.4214	−14.23	< 0.001

BDI, Beck Depression Inventory; NA, Negative Affect; SI, Social Inhibition; IS, Intrinsic Satisfcation.

**Figure 2:**
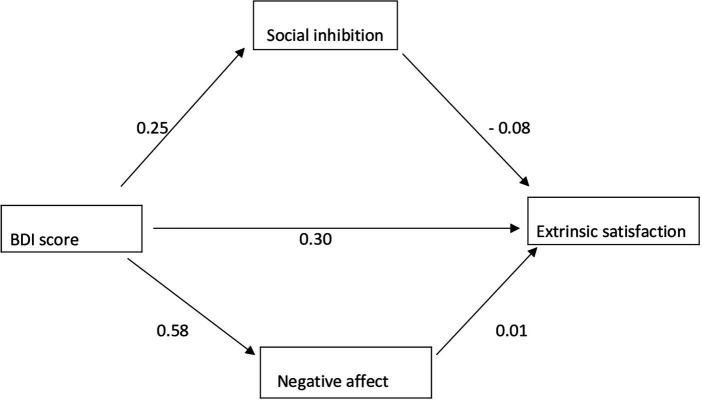
Moderator variable analysis for extrinsic satisfaction.

According to the moderation relationship conducted for the extrinsic satisfaction sub-dimension of job satisfaction, social inhibition (*β* = 0.083, SE = 0.043, *p* < 0.05) and total effect (*β* =0.298, SE = 0.022, *p* < 0.001) are the indirect effects. While the results were significant, negative affect (*β* = 0.009, SE = 0.005, *p* > 0.05) was found to have no significant effect. Although the direct effect result was significant (*β* = 0.283, SE = 0.022, *p* < 0.001), the effect level decreased when compared to the total effect when the moderator variable was included in the model. This result shows that social inhibition plays a partial moderating role in the relationship between depression and intrinsic job satisfaction, while negative affect has no role. The results are in Figure 2 and Table 3.

**Table 3:** Moderator variable analysis results for extrinsic satisfaction.

Effect	Estimate	SE	*β*	*z*	*p*
BDI⇒NA⇒ES	0.00406	0.01630	0.00550	0.249	0.803
BDI⇒SI⇒ES	−0.01558	0.00637	−0.02113	−2.446	0.014
BDI⇒NA	0.30058	0.01392	0.57597	21.590	< 0.001
NA⇒ES	0.01350	0.05423	0.00955	0.249	0.803
BDI⇒SI	0.13862	0.01725	0.25371	8.037	< 0.001
SI⇒ES	−0.11242	0.04377	−0.08327	−2.568	0.010
BDI⇒ES	−0.20889	0.02825	−0.28318	−7.394	< 0.001
BDI⇒ES	−0.22041	0.02298	−0.29880	−9.589	< 0.001

BDI, Beck Depression Inventory; NA, Negative Affect; SI, Social Inhibition; ES, Extrinsic Satisfcation.

## Discussion

The aim of this study is to investigate the moderating role of Type D personality traits (NA and SI) between the severity of depressive complaints and job satisfaction of teachers working in public schools in Turkey. To our knowledge, our study is the first to investigate the moderating role of Type D personality traits in the relationship between depressive symptoms severity and job satisfaction. According to the findings of the present study, NA and SI in the teachers have a partial moderator role between the severity of depressive complaints and intrinsic job satisfaction, and SI have a partial moderator role between the severity of depressive complaints and extrinsic satisfaction.

Many studies have reported that job satisfaction is negatively affected by psychological complaints such as depression. There are also publications showing that increasing severity of depressive complaints among teachers is associated with a decrease in job satisfaction. It is known that both the core symptoms of depression, such as anhedonia, depressed mood, and decreased self-esteem, and the vegetative symptoms (such as a decrease in sleep and energy) affect job satisfaction in teachers (32, 33, 37–40). The findings of the present study have shown that increased depressive symptom levels are correlated with lower intrinsic and extrinsic job satisfaction in the teachers. It can be said that the findings of our study are consistent with the findings of previous studies in this aspect. Of course, there may be other confounding factors that may affect the emergence of depressive complaints and job satisfaction in teachers. Therefore, future studies may help reinterpret other psychosocial factors that may cause depressive complaints in teachers and thus affect job satisfaction.

The findings of some studies have shown that personality traits are also one of the factors affecting job satisfaction. For example, there is evidence showing that job satisfaction is higher in teachers who are extroverted or have high self-efficacy (41, 42). On the other hand, it has been reported that job satisfaction is lower in teachers with predominant neurotic traits, who tend to express more negative affect (43, 44). The findings of this study showed that teachers with high negative affect and social suppression characteristics had lower job satisfaction.

Although there is no study in the literature investigating the role of type D personality structure between depression and job satisfaction, it is known that individuals with type D personality structure are more prone to depressive symptoms. For example, individuals with type D personality structure have been reported to have more suicidal thoughts along with depressive complaints (45). On the other hand, negative affect or introverted personality traits have been shown to be associated with low job satisfaction (18, 46, 47). The findings of the present study have shown that NA and SI may have a partial moderating role between depressive symptoms and internal job satisfaction. In other words, it can be said that negative affect and social inhibition play a role between depressive symptom severity and intrinsic satisfaction elements such as perception of success and recognition-appreciation. We thought that teachers with predominant negative affect and social inhibition traits might have lower intrinsic job satisfaction, related to lower self-efficacy or a more severe perception of job stress. As a matter of fact, it has been shown that type D personality structure in nurses may reduce job satisfaction due to perceived high job stress and burnout (48). In another study, type D personality was found to be associated with low self-resilience and self-esteem in nursing students (49).

Another important finding of our study is that SI has a partial moderator role between the severity of teachers’ depressive complaints and extrinsic job satisfaction. Accordingly, it can be said that social inhibition has a role between the severity of depressive complaints in teachers and extrinsic job satisfaction elements such as superior-subordinate relationship dynamics and adaptation to work conditions. In our opinion, SI characteristics of teachers who are introverts and have less social participation may be more dominant, and SI may be an important moderator between these teachers’ extrinsic job satisfaction and the severity of depressive complaints. As a matter of fact, it has been reported that low social interaction is associated with low job satisfaction (49, 50).

Although it has strengths such as having a relatively large sample size and being the first study to investigate the relationship between the type D personality structure and the severity of depressive complaints and job satisfaction, our study has some limitations. First of all, the findings are not definitive due to the cross-sectional nature of the study. The fact that the sample of the study consisted of teachers in only one city did not allow interpreting the results that could be affected by possible sociocultural differences. Additionally, the fact that all data were obtained from self-report scales can be considered a limitation.

## Conclusion

In conclusion, our findings show that the negative affect and social inhibition traits of the Type D personality structure have a moderating role between the severity of depressive complaints and job satisfaction in teachers. Training and support programs to recognize personality traits in teachers and professional psychosocial intervention programs can help increase job satisfaction. Future studies may help to understand how recognition of personality traits in teachers and professional support programs may affect job satisfaction.

## Data availability statement

The raw data supporting the conclusions of this article will be made available by the authors, without undue reservation.

## Ethics statement

The studies involving humans were approved by Van Education Research Hospital, Ethic Committee. The studies were conducted in accordance with the local legislation and institutional requirements. The participants provided their written informed consent to participate in this study.

## Author contributions

AY: Conceptualization, Formal analysis, Investigation, Methodology, Resources, Supervision, Validation, Visualization, Writing – original draft, Writing – review & editing. HK: Conceptualization, Data curation, Formal analysis, Investigation, Methodology, Writing – original draft, Writing – review & editing.
